# Methyl 6-de­oxy-6-iodo-α-d-galactoside

**DOI:** 10.1107/S1600536810022786

**Published:** 2010-06-23

**Authors:** Shivali A. Gulab, Janice M. H. Cheng, Mattie S. M. Timmer, Bridget L. Stocker, Graeme J. Gainsford

**Affiliations:** a Carbohydrate Chemistry Group, Industrial Research Limited, PO Box 31-310, Lower Hutt, New Zealand; b School of Chemical and Physical Sciences, Victoria University of Welllington, PO Box 600, Wellington, New Zealand; c Malaghan Institute of Medical Research, PO Box 7060, Wellington, New Zealand

## Abstract

In the crystal of the title compound, C_7_H_13_IO_5_, the molecules are linked by O—H⋯O hydrogen bonds, which build linkages around one screw axis of the cell. These *C*(5) and *C*(6) packing motifs expand to *R*_2_^2^(10) and C_2_^2^(11) motifs and are similar to those found for closely related compounds. The galactoside ring has a ^1^ *C*_4_ chair conformation.

## Related literature

For the synthetic details, see Dangerfield *et al.* (2009 ▶); Stocker *et al.*(2010 ▶). For related structures, see Sikorski *et al.* (2009 ▶), Robertson & Sheldrick (1965 ▶). For ring conformations see: Cremer & Pople (1975 ▶) and for hydrogen-bond motifs, see: Bernstein *et al.* (1995 ▶).
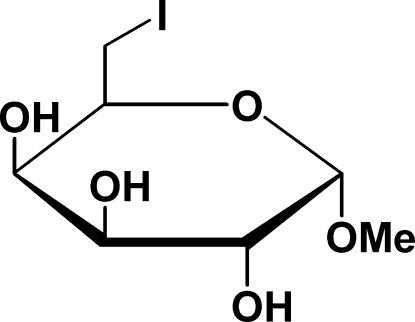


## Experimental

### 

#### Crystal data

C_7_H_13_IO_5_
*M* *_r_* = 304.08
Orthorhombic, 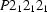

*a* = 5.7745 (2) Å
*b* = 7.9055 (3) Å
*c* = 22.1835 (7) Å
*V* = 1012.68 (6) Å^3^
*Z* = 4
Mo *K*α radiation
μ = 3.15 mm^−1^
*T* = 111 K
0.51 × 0.30 × 0.02 mm

#### Data collection

Bruker APEXII CCD diffractometer
Absorption correction: multi-scan (Blessing, 1995 ▶) *T*_min_ = 0.523, *T*_max_ = 0.747
30359 measured reflections
4062 independent reflections
3930 reflections with *I* > 2σ(*I*)
*R*_int_ = 0.033

#### Refinement

*R*[*F* ^2^ > 2σ(*F* ^2^)] = 0.021
*wR*(*F* ^2^) = 0.050
*S* = 1.06
4062 reflections
122 parameters
H-atom parameters constrained
Δρ_max_ = 1.45 e Å^−3^
Δρ_min_ = −0.78 e Å^−3^
Absolute structure: Flack (1983 ▶), 1653 Friedel pairs
Flack parameter: 0.002 (13)

### 

Data collection: *APEX2* (Bruker, 2005 ▶); cell refinement: *SAINT* (Bruker, 2005 ▶); data reduction: *SAINT* and *SADABS* (Bruker, 2005 ▶); program(s) used to solve structure: *SHELXS97* (Sheldrick, 2008 ▶); program(s) used to refine structure: *SHELXL97* (Sheldrick, 2008 ▶); molecular graphics: *ORTEP-3* (Farrugia, 1997 ▶), *Mercury* (Macrae *et al.*, 2008 ▶) and *PLATON* (Spek, 2009 ▶); software used to prepare material for publication: *SHELXL97* and *PLATON*.

## Supplementary Material

Crystal structure: contains datablocks global, I. DOI: 10.1107/S1600536810022786/lh5062sup1.cif

Structure factors: contains datablocks I. DOI: 10.1107/S1600536810022786/lh5062Isup2.hkl

Additional supplementary materials:  crystallographic information; 3D view; checkCIF report

## Figures and Tables

**Table 1:** Hydrogen-bond geometry (Å, °)

*D*—H⋯*A*	*D*—H	H⋯*A*	*D*⋯*A*	*D*—H⋯*A*
O2—H2*O*⋯O3^i^	0.84	1.90	2.7407 (15)	175
O3—H3*O*⋯O4^i^	0.84	2.01	2.8310 (16)	166
O4—H4*O*⋯O2^ii^	0.84	1.94	2.7529 (15)	163

Symmetry codes: (i) 
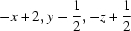
; (ii) 
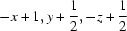
.

**Fig. 1.**
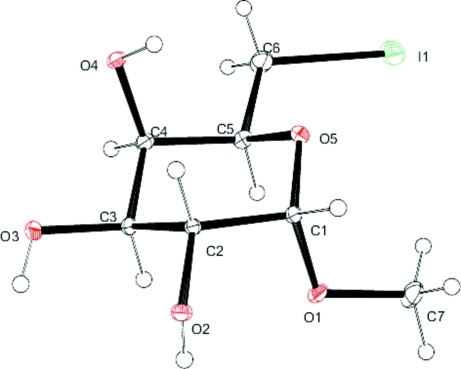
Asymmetric unit contents of (I) (Farrugia, 1997) at the 30% thermal ellipsoid level.

**Fig. 2.**
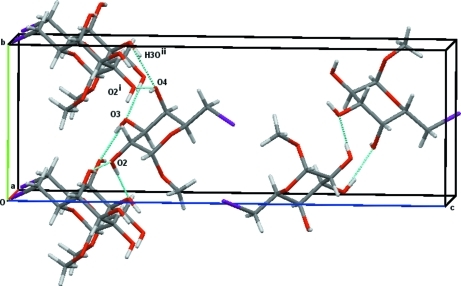
A packing view (Mercury 2.3, Macrae et al. (2008)) of the cell highlighting major hydrogen bonds (dotted). Symmetry codes: (i) 1 - x, 1/2 + y, 1/2 - z (ii) 2 - x, 1/2 + y, 1/2 - z.

## supplementary crystallographic information

### Comment

Alkyl iodoglycosides such as the title compound (I) are versatile synthetic intermediates for the introduction of a wide array of functional groups, *e.g.* amines, ethers and esters, onto a carbohydrate scaffold. In addition, the chemical transformation of iodoglycosides have led to the synthesis of a wide array of biologically important molecules (Stocker *et al.*, 2010; Dangerfield *et al.*, 2009).

The asymmetric unit of the title compound (I) contains one independent methyl 6-deoxy-6-iodo-α-D-galactoside molecule (Fig. 1). The galactoside ring (C1–C5,O5) has a ^1^C_4_ chair conformation with Q 0.5902 (14) Å, θ & φ 3.36 (15) & 279 (2)° respectively (Cremer & Pople, 1975) similar to that of the corresponding glucopyranoside 0.563 (5) Å, 4.8 (5)° & 310 (5)° (BOSLEB, Sikorski *et al.*, 2009). The absolute configurations with C1(*R*), C2(*R*), C3(S), C4(*R*), C5(S) are consistent with that expected from the synthesis.

The reported data herein (see experimental) is for the default "conventional" model: isotropic hydrogen atoms riding on their parent atoms *R*[F^2^>2σ(F^2^)] (R1), 0.0214, with a total of 122 variables. For interest, a "fully refined" model was used with isotropic hydrogen atoms and anisotropic non-hydrogen atoms giving R1 0.0211, *wR*(F^2^) (wR2) 0.0482 for all 4062 data, using 170 variables. These coordinates are available from the designated author. The su values for the non-hydrogen atoms are similar for both models (0.0017–0.0023 Å), and no significant changes are found between the structural details of the two models. As an aside, we note the dominance of the iodine scattering seen in the refinement of a model with the iodine given anisotropic, and the non-hydrogen atoms isotropic, thermal parameters with the same restrained riding H atoms giving R1, wR2 of 0.0277, 0.0618 respectively for just 62 variables!

Lattice binding is provided by O—H···O hydrogen bonds (Table 1), which build linkages around the b screw axis of the cell (Figure 2). This binding is notably similar to that observed for the bromohydrin analogue (MGALBH, Robertson & Sheldrick, 1965), and the corresponding glucopyranoside (BOSLEB). The basic motif building blocks (Bernstein *et al.*, 1995) are of C(6) & C(5) types, which combine to give ^2^*R*_2_(10) and ^2^C_2_(11) motifs. Minor packing differences are noted with the BOSLEB structure, which has similar cell dimensions, with two, rather than one C(6) motif, an additional C–H···O interaction and a different ^2^*R*_2_(10) motif.

### Experimental

Synthetic details are given in Dangerfield *et al.* (2009). The title compound was recrystallized from a solution of 10% methanol in ethyl acetate

### Refinement

One reflection (0,0,2) affected by the backstop and two clearly outlier reflections (ΔF^2^/σ(F^2^)>5) were removed from the refinment.

The methyl H atoms were constrained to an ideal geometry (C—H = 0.98 Å) with *U*_iso_(H) = 1.5*U*_eq_(C), but were allowed to rotate freely about the adjacent C—C bond. All other H atoms were placed in geometrically idealized positions and constrained to ride on their parent atoms with C—H distances of 1.00 (primary) or 0.99 (methylene) Å and O—H distances of 0.84 Å and with *U*_iso_(H) = 1.5*U*_eq_(C,*O*) (see Comment text).

### Crystal data

**Table:**

C_7_H_13_IO_5_	*F*(000) = 592
*M**_r_* = 304.08	*D*_x_ = 1.994 Mg m^−^^3^
Orthorhombic, *P*2_1_2_1_2_1_	Mo *K*α radiation, λ = 0.71073 Å
Hall symbol: P 2ac 2ab	Cell parameters from 9870 reflections
*a* = 5.7745 (2) Å	θ = 2.6–34.2°
*b* = 7.9055 (3) Å	µ = 3.15 mm^−^^1^
*c* = 22.1835 (7) Å	*T* = 111 K
*V* = 1012.68 (6) Å^3^	Plate, colourless
*Z* = 4	0.51 × 0.30 × 0.02 mm

### Data collection

**Table:**

Bruker APEXII CCD diffractometer	4062 independent reflections
Radiation source: fine-focus sealed tube	3930 reflections with *I* > 2σ(*I*)
graphite	*R*_int_ = 0.033
Detector resolution: 8.333 pixels mm^-1^	θ_max_ = 35.0°, θ_min_ = 2.7°
φ and ω scans	*h* = −8→9
Absorption correction: multi-scan (Blessing, 1995)	*k* = −12→11
*T*_min_ = 0.523, *T*_max_ = 0.747	*l* = −33→33
30359 measured reflections	

### Refinement

**Table:**

Refinement on *F*^2^	Secondary atom site location: difference Fourier map
Least-squares matrix: full	Hydrogen site location: inferred from neighbouring sites
*R*[*F*^2^ > 2σ(*F*^2^)] = 0.021	H-atom parameters constrained
*wR*(*F*^2^) = 0.050	*w* = 1/[σ^2^(*F*_o_^2^) + (0.0236*P*)^2^ + 0.4353*P*] where *P* = (*F*_o_^2^ + 2*F*_c_^2^)/3
*S* = 1.06	(Δ/σ)_max_ = 0.001
4062 reflections	Δρ_max_ = 1.45 e Å^−^^3^
122 parameters	Δρ_min_ = −0.78 e Å^−^^3^
0 restraints	Absolute structure: Flack (1983), 1653 Friedel pairs
Primary atom site location: structure-invariant direct methods	Flack parameter: 0.002 (13)

### Special details

**Table:**

Geometry. All e.s.d.'s (except the e.s.d. in the dihedral angle between two l.s. planes) are estimated using the full covariance matrix. The cell e.s.d.'s are taken into account individually in the estimation of e.s.d.'s in distances, angles and torsion angles; correlations between e.s.d.'s in cell parameters are only used when they are defined by crystal symmetry. An approximate (isotropic) treatment of cell e.s.d.'s is used for estimating e.s.d.'s involving l.s. planes.
Refinement. Refinement of *F*^2^ against ALL reflections. The weighted *R*-factor *wR* and goodness of fit *S* are based on *F*^2^, conventional *R*-factors *R* are based on *F*, with *F* set to zero for negative *F*^2^. The threshold expression of *F*^2^ > σ(*F*^2^) is used only for calculating *R*-factors(gt) *etc*. and is not relevant to the choice of reflections for refinement. *R*-factors based on *F*^2^ are statistically about twice as large as those based on *F*, and *R*- factors based on ALL data will be even larger.

### Fractional atomic coordinates and isotropic or equivalent isotropic displacement parameters (Å^2^)

**Table:**

	*x*	*y*	*z*	*U*_iso_*/*U*_eq_	
I1	0.40872 (2)	0.478714 (16)	0.504464 (5)	0.02898 (4)	
O1	0.5838 (2)	0.12645 (13)	0.34021 (5)	0.01598 (19)	
O2	0.67433 (18)	0.21293 (14)	0.22048 (5)	0.01352 (19)	
H2O	0.7581	0.1333	0.2328	0.020*	
O3	1.03501 (17)	0.45621 (14)	0.24533 (5)	0.01504 (19)	
H3O	1.0745	0.3781	0.2218	0.023*	
O4	0.77547 (18)	0.67940 (13)	0.31887 (6)	0.01447 (19)	
H4O	0.6341	0.6700	0.3106	0.022*	
O5	0.47929 (18)	0.40652 (14)	0.36225 (5)	0.01431 (19)	
C1	0.4969 (2)	0.27912 (17)	0.31726 (7)	0.0122 (2)	
H1	0.3391	0.2579	0.3003	0.018*	
C2	0.6547 (2)	0.33786 (17)	0.26630 (7)	0.0107 (2)	
H2	0.5829	0.4406	0.2478	0.016*	
C3	0.8900 (2)	0.38930 (17)	0.29162 (7)	0.0118 (2)	
H3	0.9664	0.2872	0.3093	0.018*	
C4	0.8602 (2)	0.52225 (19)	0.34128 (6)	0.0131 (2)	
H4	1.0147	0.5426	0.3603	0.020*	
C5	0.6977 (2)	0.44721 (18)	0.38928 (7)	0.0145 (2)	
H5	0.7687	0.3423	0.4064	0.022*	
C6	0.6533 (3)	0.5722 (2)	0.43929 (8)	0.0239 (3)	
H6A	0.5936	0.6788	0.4217	0.036*	
H6B	0.8013	0.5980	0.4599	0.036*	
C7	0.4228 (3)	0.0461 (2)	0.38037 (8)	0.0239 (3)	
H7A	0.4069	0.1141	0.4171	0.036*	
H7B	0.4798	−0.0668	0.3909	0.036*	
H7C	0.2717	0.0362	0.3606	0.036*	

### Atomic displacement parameters (Å^2^)

**Table:**

	*U*^11^	*U*^22^	*U*^33^	*U*^12^	*U*^13^	*U*^23^
I1	0.03654 (6)	0.02910 (6)	0.02130 (5)	0.00409 (4)	0.01094 (4)	0.00063 (4)
O1	0.0168 (4)	0.0098 (4)	0.0213 (5)	−0.0006 (4)	0.0006 (4)	0.0029 (3)
O2	0.0113 (4)	0.0111 (4)	0.0182 (5)	0.0009 (3)	−0.0005 (4)	−0.0027 (4)
O3	0.0118 (4)	0.0134 (4)	0.0199 (5)	−0.0024 (3)	0.0043 (3)	−0.0023 (4)
O4	0.0124 (4)	0.0092 (4)	0.0218 (5)	−0.0001 (3)	−0.0002 (4)	0.0013 (4)
O5	0.0124 (4)	0.0133 (5)	0.0172 (5)	0.0013 (3)	0.0022 (4)	−0.0012 (4)
C1	0.0096 (5)	0.0103 (5)	0.0168 (6)	0.0000 (4)	0.0004 (4)	0.0003 (5)
C2	0.0079 (4)	0.0101 (5)	0.0141 (6)	−0.0001 (4)	−0.0002 (4)	−0.0007 (4)
C3	0.0081 (5)	0.0109 (5)	0.0163 (6)	−0.0009 (4)	0.0005 (4)	−0.0001 (4)
C4	0.0119 (5)	0.0107 (5)	0.0167 (6)	−0.0003 (4)	−0.0016 (4)	0.0008 (5)
C5	0.0165 (6)	0.0123 (6)	0.0149 (6)	−0.0004 (5)	−0.0009 (5)	0.0002 (5)
C6	0.0341 (8)	0.0179 (7)	0.0197 (8)	−0.0040 (6)	0.0073 (6)	−0.0047 (6)
C7	0.0311 (8)	0.0190 (7)	0.0218 (7)	−0.0062 (7)	0.0037 (6)	0.0055 (5)

### Geometric parameters (Å, °)

**Table:**

I1—C6	2.1521 (18)	C2—C3	1.5252 (18)
O1—C1	1.4027 (17)	C2—H2	1.0000
O1—C7	1.436 (2)	C3—C4	1.532 (2)
O2—C2	1.4219 (18)	C3—H3	1.0000
O2—H2O	0.8400	C4—C5	1.538 (2)
O3—C3	1.4268 (17)	C4—H4	1.0000
O3—H3O	0.8400	C5—C6	1.508 (2)
O4—C4	1.4248 (17)	C5—H5	1.0000
O4—H4O	0.8400	C6—H6A	0.9900
O5—C1	1.4217 (18)	C6—H6B	0.9900
O5—C5	1.4332 (18)	C7—H7A	0.9800
C1—C2	1.524 (2)	C7—H7B	0.9800
C1—H1	1.0000	C7—H7C	0.9800
			
C1—O1—C7	111.98 (12)	O4—C4—C5	111.62 (11)
C2—O2—H2O	109.5	C3—C4—C5	107.55 (11)
C3—O3—H3O	109.5	O4—C4—H4	108.3
C4—O4—H4O	109.5	C3—C4—H4	108.3
C1—O5—C5	112.93 (11)	C5—C4—H4	108.3
O1—C1—O5	112.36 (12)	O5—C5—C6	107.77 (12)
O1—C1—C2	108.51 (11)	O5—C5—C4	109.50 (11)
O5—C1—C2	110.34 (11)	C6—C5—C4	111.12 (12)
O1—C1—H1	108.5	O5—C5—H5	109.5
O5—C1—H1	108.5	C6—C5—H5	109.5
C2—C1—H1	108.5	C4—C5—H5	109.5
O2—C2—C1	111.48 (11)	C5—C6—I1	112.39 (11)
O2—C2—C3	112.18 (11)	C5—C6—H6A	109.1
C1—C2—C3	109.92 (11)	I1—C6—H6A	109.1
O2—C2—H2	107.7	C5—C6—H6B	109.1
C1—C2—H2	107.7	I1—C6—H6B	109.1
C3—C2—H2	107.7	H6A—C6—H6B	107.9
O3—C3—C2	110.89 (12)	O1—C7—H7A	109.5
O3—C3—C4	109.21 (11)	O1—C7—H7B	109.5
C2—C3—C4	110.34 (10)	H7A—C7—H7B	109.5
O3—C3—H3	108.8	O1—C7—H7C	109.5
C2—C3—H3	108.8	H7A—C7—H7C	109.5
C4—C3—H3	108.8	H7B—C7—H7C	109.5
O4—C4—C3	112.69 (11)		
			
C7—O1—C1—O5	68.74 (15)	O3—C3—C4—O4	54.99 (14)
C7—O1—C1—C2	−168.99 (12)	C2—C3—C4—O4	−67.15 (14)
C5—O5—C1—O1	60.32 (15)	O3—C3—C4—C5	178.43 (11)
C5—O5—C1—C2	−60.91 (15)	C2—C3—C4—C5	56.30 (14)
O1—C1—C2—O2	56.83 (14)	C1—O5—C5—C6	−175.45 (12)
O5—C1—C2—O2	−179.68 (11)	C1—O5—C5—C4	63.56 (15)
O1—C1—C2—C3	−68.21 (14)	O4—C4—C5—O5	64.63 (14)
O5—C1—C2—C3	55.28 (14)	C3—C4—C5—O5	−59.47 (14)
O2—C2—C3—O3	59.55 (14)	O4—C4—C5—C6	−54.31 (16)
C1—C2—C3—O3	−175.82 (11)	C3—C4—C5—C6	−178.41 (13)
O2—C2—C3—C4	−179.31 (11)	O5—C5—C6—I1	56.19 (15)
C1—C2—C3—C4	−54.68 (14)	C4—C5—C6—I1	176.16 (10)

### Hydrogen-bond geometry (Å, °)

**Table:**

*D*—H···*A*	*D*—H	H···*A*	*D*···*A*	*D*—H···*A*
O2—H2O···O3^i^	0.84	1.90	2.7407 (15)	175
O3—H3O···O4^i^	0.84	2.01	2.8310 (16)	166
O4—H4O···O2^ii^	0.84	1.94	2.7529 (15)	163

Symmetry codes: (i) −*x*+2, *y*−1/2, −*z*+1/2; (ii) −*x*+1, *y*+1/2, −*z*+1/2.
